# New Technologies Smart, or Harm Work-Family Boundaries Management? Gender Differences in Conflict and Enrichment Using the JD-R Theory

**DOI:** 10.3389/fpsyg.2017.01070

**Published:** 2017-06-30

**Authors:** Chiara Ghislieri, Federica Emanuel, Monica Molino, Claudio G. Cortese, Lara Colombo

**Affiliations:** Work and Organizational Psychology, Department of Psychology, University of TurinTurin, Italy

**Keywords:** work-family conflict, work-family enrichment, technology-assisted supplemental work, workload, emotional dissonance, supervisory coaching

## Abstract

**Background:** The relationship between technology-assisted supplemental work and well-being outcomes is a recent issue in scientific literature. Whether the use of technology for work purpose in off-work time may have a positive or negative impact on work-family balance remains an open question and the role of gender in this relationship is poorly understood.

**Aim:** According to the JD-R theory, this study aimed to investigate the relationship between off-work hours technology assisted job demand (off-TAJD) and both work-family conflict (WFC) and work-family enrichment (WFE). Moreover, it considered two general job demands, workload and emotional dissonance, and one job resource, supervisory coaching.

**Method:** The hypotheses were tested with a convenience sample of 671 workers. Data were collected with a self-report questionnaire and analyzed with SPSS 23 and through multi-group structural equation model (SEM) (Mplus 7).

**Results:** The estimated SEM [Chi-square (510) = 1041.29; *p* < 0.01; CFI = 0.95; TLI = 0.95; RMSEA = 0.06 (0.05, 0.06); SRMR = 0.05. M = 319/F = 352] showed that off-TAJD was positively related to WFC in both subsamples; off-TAJD was positively related also to WFE only in the Male group. Workload was positively related to WFC in both Male and Female subsamples. Emotional dissonance was positively related to WFC in both subsamples and was negatively related to WFE. Supervisory coaching was strongly, positively related to WFE in both groups, and only in the Male subsample presented a low negative relationship with WFC.

**Conclusion:** This study contributes to the literature on new challenges in work-life interface by analyzing the association between off-TAJD and WFC and Enrichment. Our findings suggest it is important to pay attention to gender differences in the study of the impact of supplemental work carried out during off-work hours using technology on the work-life interface. In fact, employee perception of Company demands of being available during off-work time, with the use of technology, may have different consequences for men and women, indicating potential differences in the centrality of the working role. Practical implications, at both cultural and organizational levels, should address the use of technology during leisure time.

## Introduction

Since January 2017, the *Loi Travail* No. 2016-1088 (Ministère du Travail, de l’Emploi, de la Formation Professionnelle et du Dialogue Social, 2016) has come into effect in France and, among other things, obligates Companies with more than 50 employees to regulate the use of smartphones and emails in off-work time. For some time, several German Companies (e.g., Deutsche Telekom, Bayer, Volkswagen) have implemented regulatory policies on the use of emails for work purposes during off-work or leisure time, consistent with some studies, which showed that the intrusion of work in personal life may be a source of stress, impede the necessary recovery, and lead to consequences in terms of work-family conflict (Derks and Bakker, 2014; Derks et al., 2015). On the other hand, some studies showed that the use of smartphones might have a positive impact on the work-family balance, especially if associated with flextime. An Australian Research outlined that more than half of the respondents believed that the mobile helps them to balance their family and working lives (Wajcman et al., 2008). Therefore, it is still an open issue whether the impact of technology-assisted supplemental work (TASW) on work-family balance is positive or negative (Fenner and Renn, 2004; Derks et al., 2015). Moreover, a matter equally open is whether and how to regulate and manage the use of technology for work purposes. Using the Job Demands-Resources Model (JD-R Model, Bakker and Demerouti, 2007, 2014, 2016; Schaufeli and Taris, 2014), this study aims to contribute to the understanding of the relationship between supplemental work during off-work hours assisted by technology and work-family conflict (WFC) and enrichment (WFE), controlling for other traditional job demands and job resources and paying particular attention to gender differences. Gender is considered a crucial variable in work-family balance studies; the topic of work-family interface has always been associated with gender differences, right from the beginning of the studies on this subject, because of the differences in role salience and participation between men and women (Carr, 2002).

### New Technologies and Supplemental Work

Smartphones and tablets are the newest communication tools, which have a relevant role in work dynamics today (Rennecker and Godwin, 2005), becoming a part of everyday working life (Derks et al., 2014). This issue is part of the wider debate on the technostress that is the stress due to information excess. In other words, technostress is the stress that workers experience as a result of their use of information systems in the organizational context; it may reduce job satisfaction, commitment, productivity and produce an invasion into personal life (Ayyagari et al., 2011; Tarafdar et al., 2015). In recent years, several studies have examined the role of the use of technology for professional purposes in relation with well-being and work-family balance (Derks et al., 2015). TASW refers to the fulfillment of work tasks using information technology and telecommunications, inside or outside the home (Fenner and Renn, 2004; Derks et al., 2014), mainly through the support of smartphones and tablets.

The spread of new technologies has increased the non-standard work schedule, gradually involving evenings, nights and weekends for many workers (Derks and Bakker, 2014), in the case of formal agreements that formalize it or even without these kind of agreements. Today, the progressive sliding of work into personal life, facilitated by new technologies, not only involves workers with specific smart working agreements but also traditional workers who have defined working hours and are paid for hours worked in the office, not just for their achievements. Therefore, in this paper we focus on supplemental work during off-work hours assisted by technology (Fenner and Renn, 2004) and not on the practices that, based on formal agreements, imply that employees work out of the office for part of their expected working time (Peters et al., 2009).

This topic is particularly important in Italy since digital devices (e.g., smartphones, tablets, and laptops) ownership and their use are high in this country (We Are Social, 2017). Moreover, the huge use of the internet through many different devices is especially high among professionals: 100% of directors, managers and academic professors, 99.5% of entrepreneurs and self-employed workers, and 98.8% of office workers and teachers, according to a survey that involved a representative sample of the Italian population (Belluati, 2016). The spread of the compulsive behavior of text-message and email checking makes this behavior socially acceptable, thus making it even more widespread. Moreover, Italy is a country characterized by a weak labor market, high job insecurity and unemployment, frequent downsizing and business failures, where those people who have a job tend to intensify their workload in order to preserve it (a widespread phenomenon also in other countries, Derks and Bakker, 2014; Giunchi et al., 2016). Work intensification is both the direct consequence of the reduction of the workforce (people who stay work more) and a strategy aimed at securing work continuity (people work more to stay). Nevertheless, no systematic studies exist about the effectiveness of this strategy. Today’s organizations have high expectations of employee availability, and this factor leads employees to feel obliged to immediately answer work messages and emails, also during off-work time (Davis, 2002; Derks and Bakker, 2014).

Some studies have highlighted smartphone use advantages: interaction and collaboration between co-workers is simplified (Pica and Kakihara, 2003), work schedules are more flexible, and productivity may improve (Locke, 2005, unpublished). However, among the problematic issues, TASW is associated with constant control and supervision by others, and lack of autonomy, which generate problems particularly if people perceive the intrusion of work into the rest of their life through new technologies, which are outside of their control (Derks et al., 2014). The perception of organizations’ expectations of being constantly connected in order to respond to working requests is another problematic aspect (Bakker and Derks, 2010). Smartphone use during off-work time is associated with information overload and loss of control over information flow (Derks et al., 2014), which may be a source of stress as literature on technostress highlighted (Ayyagari et al., 2011; Tarafdar et al., 2015).

Although in certain cases the use of technology is associated with work-life balance strategies (e.g., leaving the office earlier in order to solve a family problem and conclude working activities remotely, through a phone call, text or email in the evening), it can lead to difficulties in managing the balance between work and life (e.g., Davis, 2002; Higgins and Duxbury, 2005; Jarvenpaa and Lang, 2005). Finally, for those who generally use smartphones to stay connected to their work, it can be very difficult to detach psychologically from their work (Derks and Bakker, 2014; Derks et al., 2014). This can lead to negative consequences for their well-being (Meijman and Mulder, 1998) and work-family balance (van Hooff et al., 2006). In the present study, we take into account a specific aspect of the supplemental work assisted by technology. In the framework of the JD-R Model (Bakker and Demerouti, 2007, 2014, 2016), we want to deepen the role of a specific job demand: the off-work hours technology assisted job demand (off-TAJD), that is the perceived request to use technological devices to work during off-work time.

### Work-Family Conflict and Enrichment

According to previously introduced references and in line with the boundary theory, permeable boundaries increase the spillover from work to the private domain (Ashforth et al., 2000). Problems in work-home balance are usual today and the presence of smartphones and tablets “always on,” the attitude toward continuously monitoring notifications, the tendency to react immediately to emails may have negative consequences on work-family balance (Derks et al., 2015).

Research on work-family boundaries emphasized that changes in demographics (more dual-earner or dual-career couples and single-parent families) and working conditions (more job insecurity and work intensification, blurred boundaries between work and life) has led to a lot of transformations in the work and family interaction (Ghislieri and Colombo, 2014). These changes have contributed to making workers less able to reach a satisfying work-life balance. The issues of WFC and WFE are the central constructs in this area of studies: to simplifying, several studies show that WFC is related to lower work satisfaction, higher turnover intentions, emotional exhaustion and a health impairment process (Colombo and Ghislieri, 2010; Cortese et al., 2010; Molino et al., 2016a; Ghislieri et al., 2017) while WFE is associated to high professional commitment and lower turnover intentions (Russo and Buonocore, 2012; Tummers and Den Dulk, 2013; Ghislieri et al., 2017). These findings indicate that research on WFC and WFE, intertwined with the study of the use of new technologies, can be considered an in-depth exploration of the technostress topics cited above (Ayyagari et al., 2011; Tarafdar et al., 2015).

The work-family interface is described as the process of influence between pressures and resources from the work (or family) domain and the individual’s behavior in the family (or work) domain (Bakker et al., 2011). Among the different theoretical models used to explain this process, the conflict perspective (WFC) dominated studies in the 1980 and 1990s, while the enrichment perspective (WFE) has become increasingly important in the last two decades (Ghislieri et al., 2011; Russo and Buonocore, 2012).

The WFC approach is based on the role theory (Merton, 1957) and on the role strain hypothesis (Goode, 1960). WFC has been defined as: “a form of inter-role conflict in which the role pressures from the work and family domains are mutually incompatible in some respect. That is, participation in the work (family) role is made more difficult by virtue of participation in the family (work) role” (Greenhaus and Beutell, 1985, p. 77). WFC may be time based, strain based or based on incompatible behavioral demands. Research on behavioral role conflict is infrequent and, in the present study, we only focused on time- and strain-based work to family conflict (Netemeyer et al., 1996; Derks and Bakker, 2014).

As mentioned above, in the last years, scholars have started to consider, in studies, not only WFC, but also WFE. Several authors, considering theoretical and empirical studies, points out that WFC and WFE (and related concepts like enhancement, facilitation or positive spillover) are distinct constructs (Grzywacz and Butler, 2005; Carlson et al., 2006; Ghislieri et al., 2011). About WFE, when one role enhances the quality of the other, we have an enrichment process: “WFE occurs when work experiences improve the quality of family life, and family-to-work enrichment occurs when family experiences improve the quality of work life” (Greenhaus and Powell, 2006, p. 73). The enrichment process may be instrumental, when resources move from one role to the other with a direct effect, or affective, when resources from one role create a positive affective state within this role that, consequently, fosters the performance in the other role (Greenhaus and Powell, 2006).

Scholars consider WFE a central issue for employers, employees, and organizations (Molino et al., 2013; Ghislieri et al., 2017) because research evidence shows that WFE is positively associated with job-related (Shockley and Singla, 2011), family-related (van Steenbergen et al., 2007) and health-related results (van Steenbergen and Ellemers, 2009). To enhance individual and organizational well-being, it is important to understand not only how to reduce WFC but also how to promote WFE (Bakker et al., 2011; Biggio and Cortese, 2013; Molino et al., 2013). The main determinants of WFE are job resources (Carlson et al., 2006; Bakker et al., 2010), and the role of the job demands in work-family enrichment is unclear. Despite the fact that some studies report low negative relationship between job demands and WFE (Carvalho and Chambel, 2014, there are few studies that include job demands like determinants of the WFE (Ghislieri et al., 2011). Furthermore, some authors who considered similar constructs point out that highly demanding jobs may be compatible with enrichment experiences in the presence of adequate support and autonomy (Grzywacz and Butler, 2005).

Among the different theories used to understand work-family interaction, the JD-R Theory is largely considered (Bakker and Demerouti, 2014, 2016; Schaufeli and Taris, 2014; Tement and Korunka, 2015). This theory explains health impairment and motivational processes considering two different types of working conditions as the main determinants of the processes: demands and resources. It is flexible and adaptable to a very different kind of organization and is useful not only to define implications for human resources management but also health promotion initiatives, starting from a specific identification of job demands and job resources that are relevant in a particular context.

According to the JD-R Theory, “Job demands refer to those physical, psychological, social or organizational aspects of the job that require sustained physical and/or psychological (cognitive and emotional) effort or skill and are therefore associated with certain physiological and/or psychological costs” (Bakker and Demerouti, 2007, p. 312). Job demands are not harmful by definition but they may be job stressors when great effort is required, which is not followed by adequate recovery (Meijman and Mulder, 1998). In this study, considering the research previously mentioned, we included off-TAJD in the category of job demands and formulated the following main study hypotheses:

H1a: off-TAJD is positively related to WFC.
H1b: off-TAJD is negatively related to WFE.

We tested the relationship between off-TAJD and both WFC and WFE beyond and above the effects of other traditional job demands and job resources. With regard to other job demands, we included in this study both workload and emotional dissonance. Workload is a job demand that represents the amount of tasks and activities that workers have to carry out (Schaufeli and Taris, 2014). To date, several studies have investigated this (Bakker and Demerouti, 2007; Giunchi et al., 2016; Molino et al., 2016a) and showed that it is one of the main determinants of WFC (Cortese et al., 2010; Ghislieri et al., 2012). The second job demand considered is emotional dissonance, which refers to a discrepancy between felt and displayed emotions (Zapf et al., 1999). Studies underlined that its effects can “expand” in other life domains and influence the work-family interface (Cheung and Tang, 2009; Yanchus et al., 2010; Ghislieri et al., 2012). As mentioned above, the relationship between job demands and WFE has been investigated less in literature to date, since enrichment is mainly linked to resources (Carlson et al., 2006; Bakker et al., 2010). Therefore, in this study we hypothesized a low negative relationship between job demands and WFE, mainly for those demands characterized by a strong spillover effect on the rest of life, namely emotional dissonance (Ghislieri et al., 2017).

H2a: Workload and emotional dissonance are positively related to WFC.
H2b: Workload and emotional dissonance are negatively related to WFE.

Job resources represent the second set of job characteristics and “refer to those physical, psychological, social, or organizational aspects of the job that are either/or functional in achieving work goals; reduce job demands and the associated physiological and psychological costs; stimulate personal growth, learning, and development” (Bakker and Demerouti, 2007, p. 312). Job resources are the main determinants of WFE and may reduce WFC (e.g., Voydanoff, 2004; Molino et al., 2013; Ghislieri et al., 2017). In this study, we considered supervisory coaching as a job resource; it refers to coaching behavior on the part of the supervisor that indicates respect, concern about employee needs and feelings, and help in routine tasks and skills development (Ellinger et al., 2003; Rafferty and Griffin, 2004). Previous studies found for both supervisory coaching and support a positive relationship with WFE and a negative one with WFC (e.g., O’Driscoll et al., 2003; Frye and Breaugh, 2004; Molino et al., 2013; Liu and Cheung, 2015) since supportive behavior from supervisors may generate a positive working environment (Totterdell and Holman, 2003; Emanuel et al., 2016a,b; Zito et al., 2016) and improve work-family interactions (Molino et al., 2013). In the study we hypothesized also that:

H3a: Supervisory coaching is negatively related to WFC.
H3b: Supervisory coaching is positively related to WFE.

### Gender Differences

As mentioned in the introduction, the topic of work-family interface is deeply associated with studies about gender differences (Carr, 2002). In fact, the interest about work-life interface appeared when the participation of women in the labor market gradually increased, questioning the traditional gender role division between the male breadwinner and female homemaker (Cunningham, 2008). The transformation of the attitudes of men and women in the active participation and involvement in the two major life domains (work and family) seems to vary in relation to the cohort (linked to gender role socialization processes) and to the prevalence, in different countries, of traditionalist or egalitarian gender culture (Giunchi et al., 2016), although data concerning these differences are not always convergent (Rajadhyaksha et al., 2015). In Italy, while the gender roles of men and women have become more balanced (Ghislieri and Colombo, 2014), as in other contexts (Carr, 2002), the major load still remains higher for women with regard to care work (family, children, housework), even when the occupation is challenging (Naldini and Saraceno, 2008).

Despite the work-family balance burden being especially on women, several studies have revealed similar levels of WFC and WFE perceptions between men and women in Italy (Ghislieri and Colombo, 2014), as comparably observed in other studies (Byron, 2005). In Italy, women’s participation in the labor market is a well-established phenomenon and the job search is an important opportunity for the expression of women’s identity, especially those with a high education level; the job role remains central, especially for men (Bertolini, 2013).

The role of gender as a moderator of the relationship between demands-resources and WFC and WFE has been studied but results are conflicting (Rajadhyaksha et al., 2015). In fact, some studies have reported significant differences while others have not identified specific dynamics for the two genres. Some meta-analyses have shown different relationships between WFC and potential consequences. For example, the study of Shockley and Singla (2011) noted that the negative relation between WFC and satisfaction with family life was stronger in men than in women. However, few studies investigated the gender differences in-depth in the relationship between potential antecedents and work-family interference (WFC and WFE): this aspect is central to our study. Particularly, this study investigates if the introduction of new technologies may interact with work-family balance differently for women and men. Several authors emphasized the importance to understand, in studies on work-family balance, the effect of changing in work models and practices (Amstad et al., 2011), mainly nowadays when the introduction of new technologies are influencing the transformation of gender role models (Halpern, 2005; Ford et al., 2007; Ghislieri and Colombo, 2014). The perspective from which we study this dimension is explorative; therefore, we do not define specific hypotheses with regard to this.

## Materials and Methods

### Ethics Statement

This study involved human beings through the administration of a self-report questionnaire. The Bioethical Committee of the University of Turin examined and approved the research project (14/7/2016). Since there was no medical treatment or other procedures that could cause psychological or social discomfort to participants, additional ethical approval was not required. The research was conducted in line with the Helsinki Declaration (World Medical Association, 2001), as well as the data protection regulation of Italy (Legislative Decree No. 196/2003). Participation in the research was voluntary, without receiving any reward; data collection and analysis were anonymous.

### Samples and Procedures

The study involved a convenience sample of 671 Italian workers who filled in an on-line self-report questionnaire. The reason to use the convenience sampling is due to the study’s topic of interest and to the confidentiality and the degree of anonymity that it affords its participants. In fact, the present study asked participants if their Company demands them to use technology to work during off-work hours – a demand that is not formalized in their employment contract. Using another method of survey sampling (such as choosing a particular organization to sample) employees could have been hesitant and cautious in revealing their perception about the demands from the Company to use technology to work during off-work hours. In this study, all participants were anonymous; we knew the occupational sector and job profile, not the specific organization they worked for. The cover sheet of the questionnaire explained to participants the anonymity, confidentiality and voluntariness of their participation, and the aims of the research. The informed consent was present and integrated in the on-line administration procedure.

Participants were recruited by Master degree students in Work and Organizational Psychology at the University of Turin who received training in methodological and ethical issues about questionnaire administration. Seven students volunteered to distribute the questionnaire packages to employees they know; they received instructions to collect data from employees differentiated by occupational sectors and gender. From the 700 distributed questionnaires (100 per student), 671 usable questionnaires were returned.

Among the participants, 319 were male (47.5%) and 352 were female (52.5%).

In the Male sample, 52.0% were unmarried, 42.9% married or cohabited, 5.0% separated, divorced or widowed, and 32.6% had children. Among them, 49.2% had finished high school, 36.7% had a bachelor’s or master’s degree, and 9.7% had finished elementary school. Their average age was 36.29 years (*SD* = 11.47; minimum = 19; maximum = 62). Most of the male participants had a full-time job (88.4%) and a permanent contract (72.4%). The job profile was office workers for 55.8% of male participants, blue-collar workers 23.8% and middle managers 20.4%. Participants were from different occupational sectors: 23.8% industry, 14.7% commerce, 12.9% private services, 6.3% public health, 6.3% tourism, 5.0% education and research, and 5.0% public services; the remaining participants were from other sectors. Weekly working hours were, on average, 40.02 (*SD* = 9.78; minimum = 7; maximum = 60). Mean seniority on the profession was 10.33 years (*SD* = 10.48; minimum = 1; maximum = 46).

In the Female sample, 49.7% were unmarried, 44.9% married or cohabited, 5.1% separated, divorced or widowed, and 33.5% had children. Among them, 48.9% had a bachelor’s or master’s degrees, 43.2% had finished high school, and 4.0% had finished elementary school. Their average age was 36.01 years (*SD* = 11.78; minimum = 20; maximum = 64). Most of the female participants had a full-time job (74.7%) and a permanent contract (70.5%). The job profile was office workers for 66.8% of female participants, blue-collar workers 16.5% and middle managers 16.8%. Participants were from different occupational sectors: 16.8% commerce, 13.1% private services, 12.5% public health, 10.2% education and research, 8.2% public services, 8.0% tourism, 6.8% industry, and 6.8% social sector; the remaining participants were from other sectors. Weekly working hours were, on average, 36.39 (*SD* = 9.98; minimum = 7; maximum = 60). Mean seniority on the profession was 9.38 years (*SD* = 10.58; minimum = 1; maximum = 45).

### Measures

*Work-family conflict* (WFC) was assessed with five items of the Netemeyer et al. (1996) scale (Italian version by Colombo and Ghislieri, 2008). All items were scored on a five-point scale, ranging from 1 = *never* to 5 = *always*. An example item is “Things you want to do at home do not get done because of the demands your job puts on you.” Cronbach’s alpha for the scale in this study was 0.89.

*Work-family enrichment* (WFE) was measured by three items (Ghislieri et al., 2011) scored on a Likert scale from 1 = *strongly disagree* to 5 = *strongly agree*. An example item is “At work you feel positive emotions and this helps you to be a better family member.” Cronbach’s alpha was 0.85.

*Off-work hours Technology Assisted Job Demand* (off-TAJD) was assessed with three *ad hoc* items, which asked how often, in the employee’s perception, the Company demands her/him to use technology to work during off-work hours. All items were scored on a five-point scale, ranging from 1 = *never* to 5 = *always*. An example item is “How often does your organization require you to answer phone calls and emails during off-hours?” Cronbach’s alpha was 0.94. An exploratory factor analysis (EFA) performed on a random half sample (*N* = 336) showed a one factor solution which explained 85.66% of the variance, with factor loadings equal to 0.90 for Item 1, 0.98 for Item 2 and 0.89 for Item 3. A confirmatory factor analysis (CFA) on the other half of the sample (*N* = 335) confirmed results of EFA: χ^2^(0, *N* = 335) = 0.00, *p* = 0.00, RMSEA = 0.00 (0.00, 0.00), CFI = 1.00, TLI = 1.00, SRMR = 0.00. The standardized factor loadings were 0.93 for Item 1, 0.94 for Item 2 and 0.91 for Item 3. We found that the level of off-TAJD is significantly higher for workers who receive more than 10 working e-mails per day (*M* = 6.74; *SD* = 3.93) compared with those who receive from 0 to 10 e-mails per day (*M* = 6.18; *SD* = 3.57) [*t*(664) = -1.85, *p* < 0.05].

*Workload* was assessed by four items (Bakker et al., 2004). All items were scored on a five-point scale, ranging from 1 = *never* to 5 = *always*. An example item is “You have too much work to do.” Cronbach’s alpha was 0.81.

*Emotional dissonance* was assessed with four items developed by Zapf et al. (1999). All items were scored on a five-point scale, ranging from 1 = *never* to 5 = *always*. An example item is “How often during your work do you have to display emotions which do not correspond to your inner feelings?” Cronbach’s alpha was 0.89.

*Supervisory coaching* was assessed by five items adapted from Graen and Uhl-Bien’s (1991) work. Items scored on a Likert scale from 1 = *never* to 5 = *always*. An example item is “Your supervisor informs you whether he/she is satisfied with your work.” Cronbach’s alpha was 0.91.

### Data Analysis

The statistics software SPSS 23 was used to perform descriptive data analysis in each sample separately (Males and Females). Moreover, Pearson correlations were tested in order to examine the relationships among variables, and Cronbach’s alpha coefficient was calculated to test the reliability of each scale. The analysis of variance (*t*-test for independent samples) was used to examine differences in the variables’ means between the two samples.

The psychometric characteristics of the off-TAJD scale were examined both through an EFA (Maximum Likelihood extraction) performed with SPSS Statistics 23 and through a CFA performed by Mplus 7 (Muthén and Muthén, 1998–2012). The CFA method of estimation was Maximum Likelihood (ML). The overall sample (*N* = 671) was randomly split into two subsamples homogeneous for some demographic characteristics (gender, job profile, and occupational sector): the EFA was performed on the first subsample (*N* = 336) and the CFA on the second subsample (*N* = 335).

A multi-group full structural equation model (SEM) was performed using Mplus 7 in order to test the hypothesized model and the measurement model across both Male and Female samples. The method of estimation was Maximum Likelihood (ML). According to the literature (Bollen and Long, 1993), the model was assessed by several goodness-of-fit criteria: the χ^2^ goodness-of-fit statistic; the Root Mean Square Error of Approximation (RMSEA); the Comparative Fit Index (CFI); the Tucker Lewis Index (TLI); and the Standardized Root Mean Square Residual (SRMR). Non-significant values of χ^2^ indicate that the hypothesized model fits the data. Values of RMSEA smaller than 0.05 indicate a good fit, values smaller than 0.08 indicate an acceptable fit and values greater than 1 should lead to model rejection. CFI and TLI values greater than 0.90 indicate an acceptable fit, and values greater than 0.95 indicate a good fit. The SRMR has a range from 0 to 1, with a cut-off criterion of 0.08, with higher values indicating poorer fit to the empirical data, and values lower than 0.05 indicating an excellent fit.

In order to address the common method variance issue, we conducted Harman’s single-factor test (Podsakoff and Organ, 1986; Podsakoff et al., 2003) and examined the unrotated factor solution involving all variables of interest (24 items) in an EFA. Results of this analysis showed six factors with an eigenvalue greater than one. No single factor explained a great amount of the variance (variances ranged from 5.05 to 22.93%) and the six factors combined explained 67.09%. This indicates that common method variance was not a major problem in the present study.

In order to assess the discriminant validity, using Mplus 7 we first test the measurement model, which fitted the data well [χ^2^(237, *N* = 671) = 724.88, *p* < 0.001, RMSEA = 0.06 (0.05, 0.06), CFI = 0.95, TLI = 0.95, SRMR = 0.04]. Then, we compared it with a model where the estimated correlation parameter between two of the six estimated constructs was constrained to 1.0. The chi-square difference test showed that each of the 15 constrained models (one pair of factors constrained at a time) had a significantly higher χ^2^ than the measurement model, confirming that factors are not perfectly correlated and that discriminant validity is achieved (Anderson and Gerbing, 1988). Finally, we tested multicollinearity by running the variance inflation factor (VIF) and the tolerance levels using SPSS Statistics 23. Results showed that VIF values ranged from 1.06 to 1.32 and tolerance values ranged from 0.68 to 0.94, indicating that multicollinearity problem among the variables did not exist.

## Results

Analysis of variance between the male and female samples showed a significant difference only for emotional dissonance: females perceived more emotional dissonance (*M* = 2.92, *SD* = 1.10) than males (*M* = 2.68, *SD* = 1.09) [*t*(669) = 2.83, *p* < 0.01]. The other variables, namely WFC, WFE, off-TAJD, workload and supervisory coaching, did not show any differences between Male and Female samples.

**Table 1** shows the means, standard deviations, correlations among the study variables and internal consistency of each scale, separately for Male and Female groups. All α values meet the criterion of 0.70 (Nunnally and Bernstein, 1994) as they ranged between 0.80 and 0.95. All the significant correlations between the variables were in line with the expected directions.

**Table 1:** Means, standard deviations, Cronbach’s alphas, and correlations among the study variables for Male (*n* = 319) and Female (*n* = 352).

	Male		Female							
	*M*	*SD*	*M*	*SD*	*1*	*2*	*3*	*4*	*5*	*6*
(1) WFC	2.49	0.98	2.60	0.97	*0.89/0.89*	-0.14^∗∗^	0.33^∗∗^	0.44^∗∗^	0.38^∗∗^	-0.17^∗∗^
(2) WFE	3.03	1.03	3.08	1.06	-0.11	*0.85/0.85*	0.06	-0.10	-0.29^∗∗^	0.42^∗∗^
(3) Off-TAJD	2.11	1.24	2.14	1.24	0.41^∗∗^	0.12^∗^	*0.95/0.95*	0.18^∗∗^	0.12^∗^	0.06
(4) Workload	3.46	0.85	3.47	0.90	0.40^∗∗^	-0.10	0.22^∗∗^	*0.80/0.82*	0.27^∗∗^	-0.14^∗∗^
(5) Emotional dissonance	2.68	1.09	2.92	1.10	0.36^∗∗^	-0.29^∗∗^	0.22^∗∗^	0.32^∗∗^	*0.89/0.90*	-0.35^∗∗^
(6) Supervisory coaching	3.42	1.03	3.31	1.06	-0.25^∗∗^	0.45^∗∗^	-0.05	-0.22^∗∗^	-0.31^∗∗^	*0.90/0.91*

*Correlations for the Male group below the diagonal; correlations for the Female group above the diagonal. Italic values on the diagonal are Cronbach’s α for Male/Female sample.*

*^∗^p < 0.05; ^∗∗^p < 0.01.*

WFC was positively correlated with job demands (workload, emotional dissonance, and off-TAJD) and negatively associated with job resource (supervisory coaching), across the two samples. In both samples, WFC was positively associated with workload (M: *r* = 0.40, *p* < 0.01; F: *r* = 0.44, *p* < 0.01), emotional dissonance (M: *r* = 0.36, *p* < 0.01; F: *r* = 0.38, *p* < 0.01) and off-TAJD (M: *r* = 0.41, *p* < 0.01; F: *r* = 0.33, *p* < 0.01), and negatively associated with supervisory coaching (M: *r* = -0.25, *p* < 0.01; F: *r* = -0.17, *p* < 0.01).

Work-family enrichment was negatively correlated with emotional dissonance and positively associated with off-TAJD and supervisory coaching, across samples. In both samples, WFE was negatively associated with emotional dissonance (M: *r* = -0.29, *p* < 0.01; F: *r* = -0.29, *p* < 0.01) and positively associated with supervisory coaching (M: *r* = 0.45, *p* < 0.01; F: *r* = 0.42, *p* < 0.01); workload was not significantly correlated with WFE in both groups. Only in the Male group, WFE was positively correlated with off-TAJD (M: *r* = 0.12, *p* < 0.05). WFC and WFE were not significantly correlated in both groups, as expected.

**FIGURE 1:**
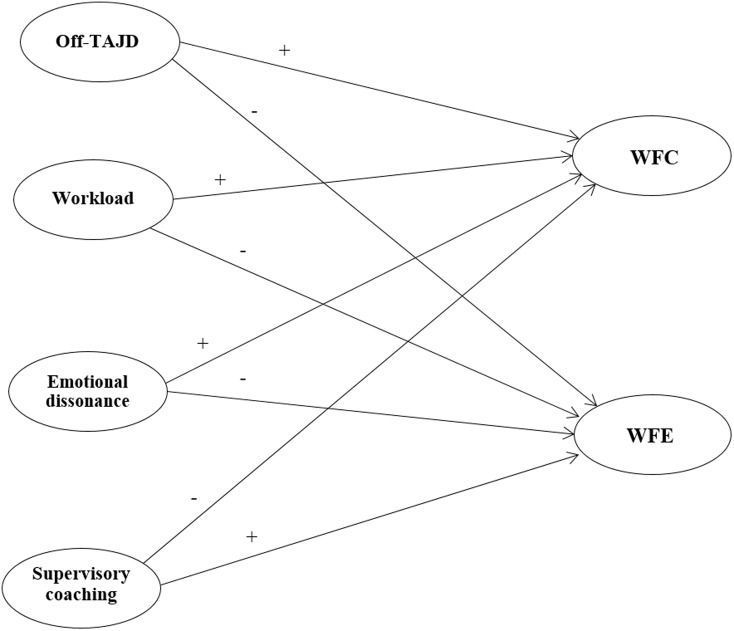
The theoretical model.

**FIGURE 2:**
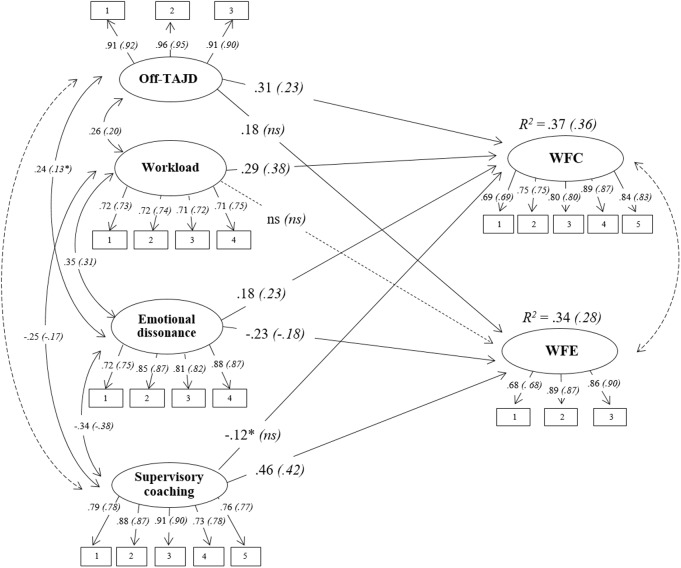
The final model (standardized path coefficients, *p* < 0.01, ^∗^*p* < 0.05). Results of the multi-group analysis: Male (*Female*). Discontinuous lines indicate non-significant relationships.

The multi-group SEM of the hypothesized model (**Figure 1**) fitted to the data well: χ^2^(510, *N*_Male_ = 319, *N*_Female_ = 352) = 1041.29, *p* = 0.00, CFI = 0.95, TLI = 0.95, RMSEA = 0.06 (90% CI 0.05, 0.06), SRMR = 0.05. **Figure 2** shows standardized parameters for both groups.

Referring to off-TAJD, it presented a significant relationship with WFC in both groups (M: β = 0.31, *p* < 0.01; F: β = 0.23, *p* < 0.01) confirming H1a. Moreover, off-TAJD had a positive relation with WFE only in Male group (M: β = 0.18, *p* < 0.01), thus H1b was confirmed only in one group.

Among the other job demands, workload had a significant relationship with WFC in both samples (M: β = 0.29, *p* < 0.01; F: β = 0.38, *p* < 0.01) and emotional dissonance was positively related to WFC across the two groups (M: β = 0.18, *p* < 0.01; F: β = 0.23, *p* < 0.01), confirming H2a. Workload did not show a relationship with WFE across the two groups; emotional dissonance was negatively related to WFE in both samples (M: β = -0.23, *p* < 0.01; F: β = -0.18, *p* < 0.01). Therefore, H2b was only partially confirmed.

With regard to job resource, supervisory coaching was negatively related to WFC only in the Male group (M: β = -0.12, *p* < 0.05), thus H3a is confirmed only in one group. Moreover, supervisory coaching was positively related to WFE across the two groups (M: β = 0.46, *p* < 0.01; F: β = 0.42, *p* < 0.01) confirming H3b in both groups. Variance of dependent variables explained by the model was 37% for WFC and 36% for WFE in the Male sample; and 34% for WFC and 28% for WFE in the Female sample. By examining the estimated model, the variables showed good item loadings in both groups.

## Discussion

The study intended to understand better the relation between the request, perceived by workers, to complete supplemental work activities by means of technological devices and work-family interface (in the work to family direction). It is the first study in literature that considered both the negative (WFC) and positive (WFE) sides of work-family interface together, and that explored gender differences within this framework. Moreover, few studies have investigated the effects of supplemental work assisted by technology in Italy so far, a country where organizations have high expectations of employee availability during off-work hours and where digital devices ownership and use is increasing (We Are Social, 2017). Finally, the study considered supplemental work assisted by technology during off-work hours as a specific job demand from the organization and not as the frequency of use of technology for work purposes.

Using the JD-R Model as a theoretical framework (Bakker and Demerouti, 2007, 2014, 2016; Schaufeli and Taris, 2014), the study highlighted a strong relationship between off-TAJD and WFC, mainly in the Male sample, according to the previous research which found, for supplemental work assisted by technology, the potential to negatively interfere with the rest of life (Higgins and Duxbury, 2005; Jarvenpaa and Lang, 2005). Findings also confirmed that workload and emotional dissonance are job demands particularly able to predict WFC, in both samples (workload especially for women). Therefore, for men, WFC is mainly a consequence of work that overcomes boundaries with a spillover effect in the family domain, in which, nowadays in Italy, men generally have a secondary role supporting women, more than really sharing tasks and responsibilities with them (Ghislieri and Colombo, 2014). For women, WFC is mainly a consequence of workload and of the resulting tiredness and fatigue.

The relationship between job demands and WFE was partially in line with study hypotheses. Among job demands considered in the study, workload did not show a relationship with WFE: the resource depletion generated by workload seems to be not such as to reduce WFE. As introduced in the theoretical framework, some authors considered the presence of workload compatible with enrichment experiences: in presence of significant resources (Grzywacz and Butler, 2005), which are the main determinants of WFE (Carlson et al., 2006), workload could have not negative effects on WFE. On the contrary, emotional dissonance was negatively related to WFE, according to previous studies (Ghislieri et al., 2017): the request to show emotions different from the ones genuinely felt could deplete emotional resources and decrease the affective enrichment perceived in the direction from work to family with, as a consequence, feelings of discomfort related to emotions management (Zapf et al., 1999; Cheung and Tang, 2009; Yanchus et al., 2010; Emanuel et al., 2014; Molino et al., 2016b). In this regard, the most interesting result was about off-TAJD: it showed a positive relationship with WFE only in the Male sample. As well as for WFC, this results might be interpreted starting from the centrality of the working role for men (Ghislieri and Colombo, 2014; Rajadhyaksha et al., 2015): being required to answer working emails and phone calls, even outside working hours, could be perceived by men as a confirmation of how their role at work is important. On the one hand, this aspect could generate conflict because of the spillover from work to the family domain, on the other, this spillover could be the consequence of the centrality of work in the person’s life and of the centrality of the person in her/his specific job position.

The study also investigated the relationship of WFC and WFE with supervisory coaching (Ellinger et al., 2003). This job resource was negatively associated with WFC only in the Male sample, according to those studies that highlighted the greater need of support by men (Rajadhyaksha et al., 2015) with regard to balancing work and family tasks. In both samples, supervisory coaching showed a positive relationship with WFE, confirming that it is an important resource able to foster the enrichment processes from work to family: the supportive relationship with a leader able to individually consider his/her employees and recognize the value of their work, might trigger processes of positive self-evaluation, recognition and self-appreciation which, in turn, could positively influence the rest of their life (Liu and Cheung, 2015; Ghislieri et al., 2017).

Despite several studies considered the use of technologies as a potential cause of stress, in line with literature on technostress (Ayyagari et al., 2011; Tarafdar et al., 2015), this study highlighted that consequences of technology assisted supplemental job demands are a more complex and multifaceted issue. Particularly, considering the work-family interface, the study confirmed previous evidences about the relationship between supplemental work assisted by technology and WFC (Davis, 2002; Higgins and Duxbury, 2005; Jarvenpaa and Lang, 2005), which is a dimension related to discomfort and stress (Ghislieri and Colombo, 2014). Moreover, this study is the first that considered both positive and negative outcomes, since it investigated also the relationship with WFE. Results showed that, for men, off-TAJD may have also positive effects on enrichment; this could improve their availability to be on-call and responsive, by means of technology, also during off-work hours.

Therefore, this research contributed to the branch of studies that try to identify variables able to influence the relationships between work-related use of technology and both work-family balance and well-being. For example, in a recent study Derks et al. (2016) showed that for people who prefer integration to segmentation (like boundary management preferences), work-related smartphone use during off-job time is linked with better family role performance by the reduction of the work-family conflict.

### Limitations and Future Studies

As all research with a cross-sectional approach, this study did not permit establishing causality relations between variables (Podsakoff et al., 2012). Further studies, using longitudinal design, should examine the causal relationship between off-TAJD, workload, emotional dissonance and supervisory coaching as determinants, and WFC and WFE as consequences. Moreover, the sampling procedure (convenience sampling) adopted in this study poses constraints on the extent to which the study’s findings can be generalized.

A second limitation is the use of self-reported questionnaires that can potentially contaminate results, since observed relationships may be artificially overestimated because of the respondents’ tendency to answer in a coherent way. Nevertheless, self-reported data seemed to be the most appropriate approach in our study since it evaluated workers’ subjective perceptions of job demands, job resources, WFC and WFE.

Another limitation is the questionnaire online administration procedure. The use of Internet represents the opportunity of having a large group of individuals and lower costs associated with collecting data. At the same time, it is a limitation (Kraut et al., 2004) because it is difficult to control the study environments and Web users have different types of hardware, software, and Internet connections. Furthermore, extraneous or temporary factors could influence responses.

Moreover, according to the JD-R model (Bakker and Demerouti, 2014), future studies should investigate the buffering role of some resources in the relationship between demands and WFC and WFE (Molino et al., 2015). In particular, as for the relationship between off-TAJD and WFC, the moderating role of resources such as job autonomy and transformational leadership could be investigated.

A further study limitation is the absence of objective data, including the number of emails and phone calls received during off-work time. This kind of data could be detected better in studies within specific working contexts. The present study, considering a heterogeneous sample with different job occupations, organizations and sectors, intended to observe the relationships among variables in a general and cross way. In the future, more specific studies could focus on the specificity and distinctiveness of particular working contexts, also considering the influence of the organizational culture (Schein, 2010).

Finally, future studies could integrate the quantitative research method with qualitative ones, in order to understand better the meaning of off-TAJD in employee opinions, what mechanisms can amplify its influence on the rest of life, and what processes of perception and meaning construction around these topics men and women at work apply, taking into account both the organizational culture and national culture.

## Conclusion

The use of technology for work purposes outside of working hours, in leisure time, can play a role in the work-life balance, and the study’s results suggested that, in most cases, limiting its use could reduce the work-life balance problem. At the same time, the study highlighted how, in some cases, the use of technology is also associated with work-family enrichment. Furthermore, this concerns men for whom the work role represents a central aspect of identity, especially in some cultural contexts.

In line with previous work (Derks et al., 2014), these results suggested that the “always on” approach, typical of some organizations, which seemed to require workers to be always “online,” might generate work-family balance problems, although in some cases, it could be perceived by men as an element of role gratification.

It is essential that organizations and, in particular, people in positions of responsibility, clearly communicate expectations about workers’ behavior: the use of technology for supplemental work needs to be an element clearly addressed and recognized in work, and the impact and the consequences on the rest of life must be monitored. In cases where the use of technology during off-work time is not strictly indispensable, it needs to be regulated or reduced, or even avoided as has already been done in some cases. Moreover, it is important to consider that the inability to disengage from work during leisure time may interfere with opportunities for adequate recovery (Derks and Bakker, 2014), with potential negative consequences for well-being (Meijman and Mulder, 1998; Sonnentag and Fritz, 2007) and work-family balance itself (van Hooff et al., 2006; Derks and Bakker, 2014; Molino et al., 2015).

However, it is important to underline that organizational culture, expected behavior and leadership style, and other job characteristics, are highly influential on the work-family interface (Wajcman et al., 2008): smartphones are only a way that may be used to exert influence, positive or negative. Taking this into account, it is crucial to understand what explicit or implicit messages employees receive about their “availability” in off-work time through the use of technology (e.g., leaders example behavior, human resources development systems etc.). In this process, the role of leadership is crucial (Ghislieri and Gatti, 2012) both in terms of what is stated and of behavior truly put in place.

## Author Contributions

All authors (CG, FE, MM, CC, LC) contributed to this work. CG wrote the introduction and designed the study. CG and LC developed the method and collected the data, wrote the manuscript and received substantial input from co-authors. CC supervised the research team and contributed to the introduction and discussion sections of the manuscript. MM and FE contributed to methods and performed data analysis. All authors contributed to the conclusion and practical implications section of the manuscript, and approved the final version of the manuscript for submission.

## Conflict of Interest Statement

The authors declare that the research was conducted in the absence of any commercial or financial relationships that could be construed as a potential conflict of interest.
